# Oroxyloside A Overcomes Bone Marrow Microenvironment-Mediated Chronic Myelogenous Leukemia Resistance to Imatinib via Suppressing Hedgehog Pathway

**DOI:** 10.3389/fphar.2017.00526

**Published:** 2017-08-11

**Authors:** Xiaobo Zhang, Yicheng Liu, Lu Lu, Shaoliang Huang, Youxiang Ding, Yi Zhang, Qinglong Guo, Zhiyu Li, Li Zhao

**Affiliations:** ^1^State Key Laboratory of Natural Medicines, Jiangsu Key Laboratory of Carcinogenesis and Intervention, China Pharmaceutical University Nanjing, China; ^2^Jiangsu Key Laboratory of Drug Design and Optimization, China Pharmaceutical University Nanjing, China

**Keywords:** microenvironment, cell co-culture, drug resistance, hedgehog pathway, BCR-ABL, Oroxyloside A

## Abstract

Imatinib (IM), as first inhibitor of the oncogenic tyrosine kinase BCR-ABL, has been widely used to treat chronic myeloid leukemia (CML) for decades in clinic. However, resistance to IM usually occurs in CML patients. The bone marrow (BM), as the predominant microenvironment of CML, secretes an abundant amount of cytokines, which may contribute to drug resistance. In current study, we utilized *in vitro* K562 co-culture model with BM stroma to investigate IM resistance. As a result, co-culturing of K562 with BM stroma was sufficient to cause resistance to IM, which was accompanied with the activation of hedgehog (Hh) signaling pathway and upregulation of BCR-ABL as well as its downstream proteins like phosphorylated Akt, Bcl-xL and survivin, etc. On the other hand, oroxyloside A (OAG), a metabolite of oroxylin A from the root of *Scutellaria baicalensis Georgi*, which had low toxic effect on K562 cells, was able to sensitize K562 cells co-cultured with BM stroma to IM treatment *in vitro* and *in vivo*. We observed that OAG suppressed Hh pathway and subsequently nuclear translocation of GLI1, followed by downregulation of BCR-ABL and its downstream effectors, thus facilitating IM to induce apoptosis of K562 cells. Together, BM microenvironment rendered K562 cells drug resistance through activating Hh signaling, however, OAG could overcome IM resistance of CML cells through inhibiting Hh-BCR-ABL axis *in vitro* and *in vivo*.

## Introduction

Chronic myeloid leukemia (CML) developed in most patients due to the expression of hybrid BCR-ABL protein resulted from the genetic rearrangement formed reciprocal translocation between chromosomes 9 and 22. The BCR-ABL protein harbored with constitutive tyrosine kinase activity, which lead to the enhanced proliferation, resistance to apoptosis and altered adhesion. Although imatinib (IM) was first developed as an inhibitor of BCR-ABL kinase for treatment of CML, the development of drug resistance became obstacle of its success in clinic, which was attributed to not only BCR-ABL gene amplification and kinase domain mutation, but also low oral bioavailability, high plasma-protein binding efficiency and altered drug efflux and/or influx mechanisms (Apperley, 2007; Milojkovic and Apperley, 2009). Thus, overcoming IM resistance has attracted more attentions during treatment of CML.

The bone marrow (BM) microenvironment consists of a cellular compartment and a non-cellular compartment including the extracellular matrix (ECM) and soluble factors (cytokines, growth factors, and chemokines), plays a vital role in cancer initiation, progression, drug resistance and relapse of hematologic malignancies (Manier et al., 2012). The soluble factors derived from BM stroma cells (BMSC) have contributed to the self-renewal, survival, and resistance to chemotherapy of leukemia cells (Nefedova et al., 2003; Dazzi et al., 2006; Yamamoto-Sugitani et al., 2011). HS-5, an immortalized human BMSC-derived cell line, potently secretes various soluble factors involved in initiating expansion of CD34^+^ cells, among which granulocyte-macrophage-colony-stimulating factor (GM-CSF) and IL-6 have been previously reported to regulate Hedgehog (Hh) pathway (Roecklein and Torok-Storb, 1995; Merchant et al., 2010; Su et al., 2013). The Hh signaling pathway is initiated by the binding of Hh ligand like Sonic hedgehog (Shh) to Patched (PTCH), resulting in a second transmembrane protein Smoothened (SMO) activation and GLI1 translocation to nucleus, where it activates target genes transcription (Pasca di Magliano and Hebrok, 2003; Mar et al., 2011; Ryan and Chiang, 2012). Recent publications have shown that Hh pathway activation is closely associated with the expansion of leukemia stem cells (LSCs) (Zhao et al., 2009), and targeting Hh pathway could overcome IM resistance in BCR–ABL positive CML (Zeng et al., 2015). Collectively, these evidences raised the possibility that BM microenvironment activated Hh pathway could render drug resistance in CML.

Oroxylin A (OA) is one of naturally bioactive flavonoids derived from *Scutellaria baicalensis Georgi*, exhibiting the activity of anti-inflammation, antibacterial infections, antiviral and anticancer (Li and Chen, 2005). However, oroxyloside A (OAG) would be the predominant form present in the systematic circulation during the oral administration of OA (Li et al., 2011) and has been barely investigated. Recently we showed OAG prevented dextran sulfate sodium-induced experimental colitis in mice (Wang et al., 2016). Nevertheless, it is still unknown whether OAG could function as OA did previously in term of overcoming drug resistance (Zhu et al., 2012, 2013; Wang Y. et al., 2014; Zhao et al., 2015).

In current study, we sought to evaluate the role of OAG in overriding drug resistance. To this end, we generated BM microenvironment-mediated IM resistance model by co-culturing K562 cells with HS-5 cells. Although the role of Hh signaling pathway in CML drug resistance has been reported (Zeng et al., 2015), we provided further evidence showing that BM mimicked by HS-5 cells contributed to Hh signaling pathway activation, and subsequently K562 cell resistance to IM treatment. More importantly, OAG could suppress Hh signaling pathway and BCR-ABL upregulation, thus facilitating IM to cause K562 cell apoptosis in co-cultured system.

## Materials and Methods

### Chemistry

All reagents were from commercial sources. With tetramethylsilane (TMS) as internal standard, the ^1^H NMR was recorded on Bruker AV-300 apparatus by using deuterated solvents. HR-MS was collected on Agilent technologies 6520 Accurate-Mass Q-TOF LC/MS instruments. Every targeted compound was purified via silica gel (60Å, 70–230 mesh) column chromatography.

**methyl (2*S*, 3*S*, 4*R*, 5*R*, 6*R*)-3, 4, 5, 6-tetrahydroxytetrahydro-2*H*-pyran-2-carboxylate**

Material A (35.2 g, 0.20 mol) was dissolved in CH_3_OH (200 ml) and the pH was adjusted to 8 with 1 M/L CH_3_ONa at 0°C temperature. The mixture was stirred for 6 h at room temperature maintaining the pH value of 8. Monitored by TLC, the reaction was completed and provided **compound B** 37.5 g, yield: 90%.

**(2*R*, 3*S*, 4*S*, 5*R*, 6*R*)-6-(methoxycarbonyl)tetrahydro-2*H*-pyran-2, 3, 4, 5-tetrayl tetraacetate**

To the mixture of acetic anhydride (200 ml) and **compound B** (37.9 g, 0.18 mol), HClO_4_ (1 ml) was added dropwise. The reaction was stirred overnight. By replenishing HClO_4_ (0.5 ml), the mixture was reacted for 4–5 h sequentially and affording **compound C** 14.6 g, yield: 21%.

**(2*S*, 3*R*, 4*R*, 5*S*, 6*S*)-2-bromo-6-(methoxycarbonyl)tetrahydro-2*H*-pyran-3, 4, 5-triyl triacetate**

**Compound C** (9.0 g, 0.023 mol) was added to HBr/acetic acid (90 ml) at low temperature, stirring for 1 h at 0°C and 3 h at room temperature. Then the system was extracted by water (40 ml) and dichloromethane (50 ml), and the dichloromethane layer was adjusted to neutral by saturated NaHCO_3_ (50 ml). After filtrated, the precipitate was dried and gave **Compound D** 6.03 g, yield: 66%.

**(2*S*,3*R*,4*R*,5*S*,6*S*)-2-((5-hydroxy-6-methoxy-4-oxo-2-phenyl-4H-chromen-7-yl)oxy)-6-(methoxycarbonyl)tetrahydro-2*H*-pyran-3, 4, 5-triyl triacetate**

**Compound D** (5.0 g, 0.013 mol), OA (2.3g, 0.0081 mol) and CaSO_4_ (2.3g, 0.0169 mol) were dissolved into quinoline (60 ml), then, Ag_2_CO_3_ was added. The mixture was reacted in dark at room temperature overnight. After that CHCl_3_ (60∼70 ml) was added to the system, whose filtrate was washed with dilute sulphuric acid till there was no temperature rising, and CHCl_3_ layer was collected and purified as **Compound E** 1.8 g, yield: 28%.

**Compound E** (1.8 g, 0.00388 mol) was dissolved in acetone (36 ml), 1 M/L NaOH was added dropwise for 30–40 min at a low temperature, after acetone was eliminated, the system should be adjusted to pH 3–4 until there was solid precipitating, followed by filtrating and drying, the final product **Oroxyloside A (OAG)** was obtained 1.1g, yield: 71%. ^1^H NMR (300 MHz, DMSO-*d_6_*) δ 3.35–4.09 (sugar H), 3.78 (3H, s, OCH_3_), 5.35 (1H, d, J = 6.5 Hz, H), 7.06 (1H, s, H), 7.13 (1H, s, H), 7.60–7.62 (3H, m, H), 8.08–8.11 (2H, m, H), 12.8 (1H, s, 5-OH). HRMS (ESI): m/z, calculated for C_22_H_21_O_11_, 461.1078 (M + H)^+^, found 461.1075, and the purity was over 99% by HPLC (Supplementary Figures S1, S2).

### Biochemical Reagents

Primary antibodies against Shh, GLI1, BCR-ABL, BCL-XL, Caspase 3, 7 and 9 were purchased from Cell Signaling Technology (Beverly, MA, United States). Primary antibodies against GAPDH, Tubulin α/β, PI3K, BAD, and p-BAD (Ser136) were obtained from Bioworld (Dublin, OH, United States). Primary antibodies against AKT1/2/3, p-AKT1/2/3 (Ser473), and Survivin were purchased from Santa Cruz Biotechnology, Inc. (Santa Cruz, CA, United States). IRDye^TM^ 800 conjugated secondary antibodies were obtained from Rockland Inc. (Philadelphia, PA, United States). FITC-conjugated CD13 antibody was bought from eBioscience (San Diego, CA, United States). GLI1 small interfering RNA (siRNA) and GANT61 was purchased from Santa Cruz Biotechnology. MTT (3-(4, 5-dimethylthiazol-2-yl)-2, 5-diphenyl tetrazolium bromide) was purchased from Fluka Chemical Corp. (Ronkonkoma, NY, United States).

### Cell Culture

The human CML K562 cell line was purchased from the cell bank of Shanghai Institute of Biochemistry and Cell Biology, Chinese Academy of Sciences. The human stromal cell line HS-5 was purchased from the American Type Culture Collection (CRL-11882). Cells were cultured in RPMI-1640 medium (Gibco, Invitrogen Corporation, Carlsbad, CA, United States) supplemented with 10% fetal bovine serum (FBS) (Gibco), 100 U/ml of penicillin, and 100 U/ml of streptomycin in a humidified CO_2_ (5%) incubator at 37°C.

For cell co-culture system, HS-5 cells (3 × 10^5^ /well) were first seeded in 6-well plates overnight. K562 cells were placed onto the porous trans-well inserts (0.4 μm, MILLIPORE, United States) which allowed them to share the soluble growth factors from HS-5 cell layers. All treatments were performed after co-culturing for 24 h.

### Cell Growth Assay

Cell proliferation was evaluated with Ki67 Detection Kit (KeyGen Biotech, Nanjing, China) according to the manufacturer’s instructions. The photograph was taken under light microscope (Nikon Instruments, Lewisville, TX, United States). Ki67 index was the proportion of positive staining cells in total cells.

Anchorage-independent cell growth *in vitro* was assessed by Soft Agar Colony Formation Assay. Briefly, cells were collected and seeded in six-well plates at 15,000 cells/well in 2×RPMI-1640 medium containing 0.8% agar (Oxoid, Basingstoke, United Kingdom) and 2 × FBS over a 1.2% agar layer. The plates were maintained for 21 days with humidified CO_2_ (5%) at 37°C. The colonies were photographed. For each group, all formed colonies were counted out under inverted microscope equipped with a color camera (Nikon Instruments). The percentage was calculated when set control group as 100.

Cell viability was measured by MTT assay as previously reported (Wang Y. et al., 2014). The inhibition ratio (%) was calculated as [(A_control_ — A_treated_/A_control_ × 100]. A_treated_ and A_control_ are the average absorbance of three parallel wells from each group.

### Cell Apoptosis Assay

DAPI staining was employed to detect cell apoptosis. Briefly, cells were fixed with 4% paraformaldehyde (PFA) for 30 min, washed thrice with cold PBS for 5 min and incubated with 0.1%Triton X-100 for 10 min, followed by staining with DAPI (1 mg/ml) for 15 min. The pictures were taken by fluorescence microscopy (Olympus Corporation, Tokyo, Japan).

Cell apoptosis was also evaluated with Annexin V/PI Cell Apoptosis Detection Kit (KeyGen Biotech) according to the manufacturer’s instructions. The stained cells were subjected to FACSCalibur flow cytometer (BD, San Jose, CA, United States) equipped with Cell-Quest software. The early apoptotic cell was Annexin V positive and PI negative, whereas late apoptotic one was both Annexin V and PI positive.

### Nuclear Protein Fractionation

Cells were collected and washed with PBS. Fractionation of nuclear and cytoplasmic protein was performed with Nuclear/Cytosol Fractionation Kit (BioVision, Mountain View, CA, United States) following the manufacturer’s protocol. Then the nuclear extract was subjected to western blot analysis.

### Western Blot Analysis

The Western blot was conducted as described previously (Chen et al., 2013). All data were analyzed by Odyssey Scanning System equipped with densitometry detection software (LI-COR, Inc., Lincoln, NE, United States).

### RNA Isolation and Real-time PCR

Total RNA was extracted by Trizol reagent (Invitrogen, Carlsbad, CA, United States). One microgram of RNA was reverse transcribed using cDNA synthesis kit (TaKaRa, Dalian, China) according to the manufacturer’s instruction. Real-time PCR was conducted using Applied Biosystems 7500 System (Carlsbad, CA, United States) by combining FastStart Universal SYBR Green Master (Roche) with specific primers as follows: *GLI1*, forward primer, 5′-TTCCTACCAGAGTCCCAAGT-3′, and reverse primer, 5′-CCCTATGTGAAGCCCTATTT-3′; *BCR-AB*L, forward primer, 5′-TGACCAACTCGTGTGTGAAACTC-3′; and reverse primer, 5′-TCCACTTCGTCTGAGATACTGGATT-3′; *GAPDH*, forward primer, 5′-CCACCCATGGCAAATTCCATGGCT-3′, and reverse primer, 5′-TCTAGACGGCAGGTCAGGTCCACC-3′. Data were analyzed according to the comparative Ct method, and the expression of target gene was normalized to *GAPDH* expression levels in each sample.

### RNA Interference

K562 cells were plated on 6-well plates overnight. Specific siRNA against GLI1 gene was transiently transfected into cells at a final concentration of 100 nmol/L using PepMute siRNA & DNA Transfection Reagent (SignaGen, Ijamsville, MD, United States) according to the manufacturer’s instructions. After 24 h transfection, gene silencing was measured by real-time PCR and western blot analysis.

### Immunofluorescence Confocal Microscopy

K562 cells were collected and applied to the glass coverslips after treatment, and then were subjected to immunofluorescence as described previously (Zhao et al., 2015).

### Animal Study

Animal studies were carried out in accordance with the regulations of the China Food and Drug Administration (CFDA) of China on Animal Care. This study was reviewed and approved by Animal Care Use Committee of Jiangsu Province (Animal authorization reference number: SYXK2012-0035). Twenty female NOD/SCID immune-deficient mice (aged 6–9 weeks) from Shanghai SLAC Laboratory Animal Company Limited were raised in air-conditioned pathogen-free rooms under controlled lighting (12 h per day) and fed with sterilized laboratory food and water. K562 cells (5 × 10^6^) were injected into mouse via tail vein (16 mice in total). After 1 week, the mice inoculated with K562 were randomly divided into four groups: (a) untreated group as a negative control; (b) OAG monotherapy (30 mg/kg); (c) IM monotherapy (200 mg/kg); (d) OAG combined with IM. The non-injected mice (4 mice) were set as blank control group. OAG was given intravenously once every 3 days, and IM was administered orally once every day. Mice were sacrificed 30 days later and BM, peripheral blood (PB), and spleens were collected. Spleen coefficient was calculated as (W_spleen_/W_body_) × 100. W_spleen_ and W_body_ is spleen weight and body weight of mouse, respectively. The expression of CD13 was examined by flow cytometry (BD) to evaluate K562 cell proliferation *in vivo*.

### Immunohistochemistry

Protein expression in spleen was assessed using specific antibodies together with Ultra-Sensitive^TM^ SP kit (Maixin-Bio, Fuzhou, China) as described previously (Wang H. et al., 2014).

### Statistical Analysis

Statistical analysis of multiple group comparisons was performed by one-way analysis of variance (ANOVA) followed by the Bonferroni *post hoc* test. Comparisons between two groups were analyzed using the two-tailed Student’s *t-*test. *P-*value < 0.05 was considered statistically significant.

## Results

### Co-culture Protects K562 Cells from Cell Death Induced by IM

**FIGURE 1:**
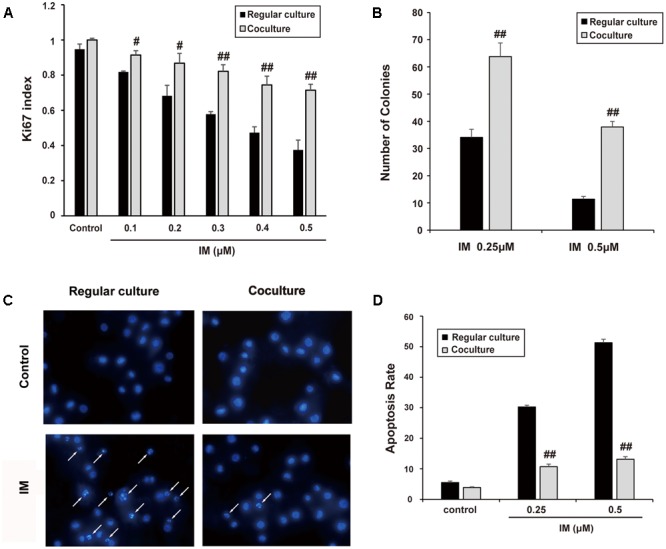
Cell co-culture rendered K562 cells resistant to IM. K562 cells were cultured alone or co-cultured with HS-5 cells for 12 h and then treated IM as indicated for 36 h. Cells were subjected to Ki67 cell proliferation detection, Soft agar assay, DAPI and Annexin V/PI staining assay as described in Section “Materials and Methods.” **(A)** Cell growth indexes were calculated by counting Ki67 positive staining of K562 cells. **(B)** Soft agar colony assay was performed and all formed colonies of regular cultured and co-cultured cells in each well were quantified when treated with indicated concentration of IM. Results represent mean values of three independent experiments ± SD. **(C)** Representative photos of DAPI staining were present and nuclear condensation was labeled as white arrows. **(D)** Regular cultured and co-cultured cells treated with indicated concentration of IM were subjected to Annexin V/PI double-staining and then analyzed by flow cytometry. The apoptotic rate of cells was represented as mean ± SD for three independent experiments. (^#^*p* < 0.05 and ^##^*p* < 0.01 compared with regular culture).

K562 cells were co-cultured with HS-5 cells as described in part of Section “Materials and Methods.” Then, cell growth and apoptosis of K562 cells was examined in the presence of IM. As shown in **Figure 1A**, IM could suppress K562 cells growth in a dose-dependent manner, whereas co-cultured cells seemed to be more refractory to IM treatment compared with regular culture as evaluated by Ki67 staining. In addition, the clonogenic assay was carried out to determine the ability of K562 cells to divide and form colonies. As a result, co-cultured K562 cells formed more colonies following IM treatment compared to regular-cultured cells (**Figure 1B** and Supplementary Figure S3A). Consistently, we observed that nuclear condensation (**Figure 1C**) and apoptosis events (**Figure 1D** and Supplementary Figure S3B) of K562 cells induced by IM were significantly attenuated due to co-culturing with HS-5 cells. These data suggested not only could co-culturing promote K562 cell proliferation, but also protect it from apoptosis caused by IM, which at least in part contributed to the acquirement of drug resistance.

### Enhanced Hh Signaling and BCR-ABL Upregulation Associates with Co-cultured K562 Cells

**FIGURE 2:**
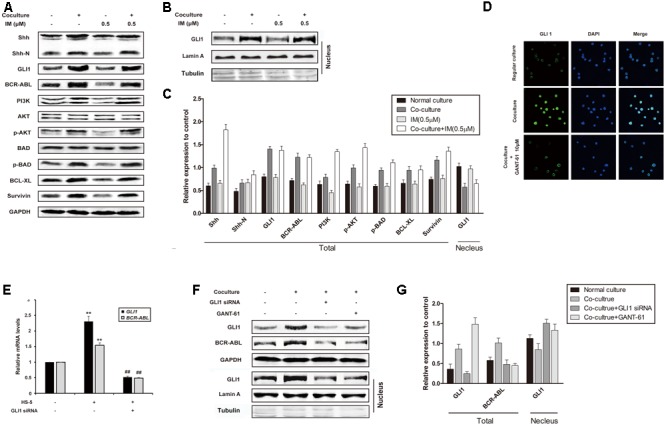
Cell co-culture up-regulated BCR-ABL via activating hedgehog pathway. **(A)** Regular cultured and co-cultured K562 cells were maintained for 24 h followed by treatment with IM for another 36 h. The lysates were subjected to western blot analysis using antibodies against Shh, Shh-N, GLI1, BCR-ABL and its downstream proteins as indicated. **(B)** Cells were cultured and treated as described above. Nuclear proteins were isolated and subjected western blot analysis using GLI1 antibody. Lamin A and Tubulin protein expression was set as nuclear and cytoplasmic marker, respectively. **(C)** The protein level was quantified and the data represented mean ± SD of three independent experiments. **(D)** K562 cells were cultured alone or co-cultured with HS-5 cells for 24 h, then treated with GANT-61 for 24 h. Immunofluorescence was performed with GLI1 antibody. DAPI was used to stain nuclei. **(E)** Control and Specific siRNA against *GLI1* was transfected into regular cultured and co-cultured K562 cells as described in Section “Materials and Methods.” Total RNA was isolated and 1 μg RNA was subjected to quantitative real-time reverse transcription-PCR for analysis of *GLI1* and *BCR-ABL* mRNA levels. Fold changes were normalized to GAPDH, and represented as mean ± SD of three replicates. **(F,G)** Cells were treated with GANT-61 and *GLI1* siRNA as described in **(D,E)**. Western blot analysis was carried out to analyze the expression of GLI1 and BCR-ABL. (^##^*p* < 0.01 compared with adjacent group and ^∗∗^*p* < 0.01 compared with regular culture).

To rule out the role of Hh signaling pathway in co-culture-mediated IM resistance, we examined the expression of important proteins of this pathway. As shown in **Figures 2A–C**, enhanced expression of Shh and GLI1 in whole lysate and nuclear extract was observed in co-cultured K562 cells, which was not affected by the addition of IM. Interestingly, BCR-ABL and its downstream targets such as PI3K, p-AKT, p-BAD, Bcl-xL and Survivin, were also up-regulated in co-culture cells with or without IM treatment, compared to that of regular culture cells (**Figure 2A**). These data indicated that Hh signaling pathway could be activated by cytokines from HS-5 cells, accompanied with IM resistance. As expected, nuclear translocation of GLI1 in co-cultured K562 cells was blocked by GANT-61 (Hyman et al., 2009), which was a small-molecule inhibitor of GLI1-mediated transcription (**Figure 2D**). Moreover, silencing of GLI1 or pharmacologic inhibition using GANT-61 was accompanied with BCR-ABL down-regulation at both mRNA and protein level in co-culture system (**Figures 2E–G**). Together, these results suggested that Hh signaling activation subsequently resulted in aberrant upregulation of BCR-ABL and downstream targets in the co-culture model, which might contribute to IM resistance of K562 cells.

### OAG Sensitizes K562 Cells to IM in Co-culture Model

**FIGURE 3:**
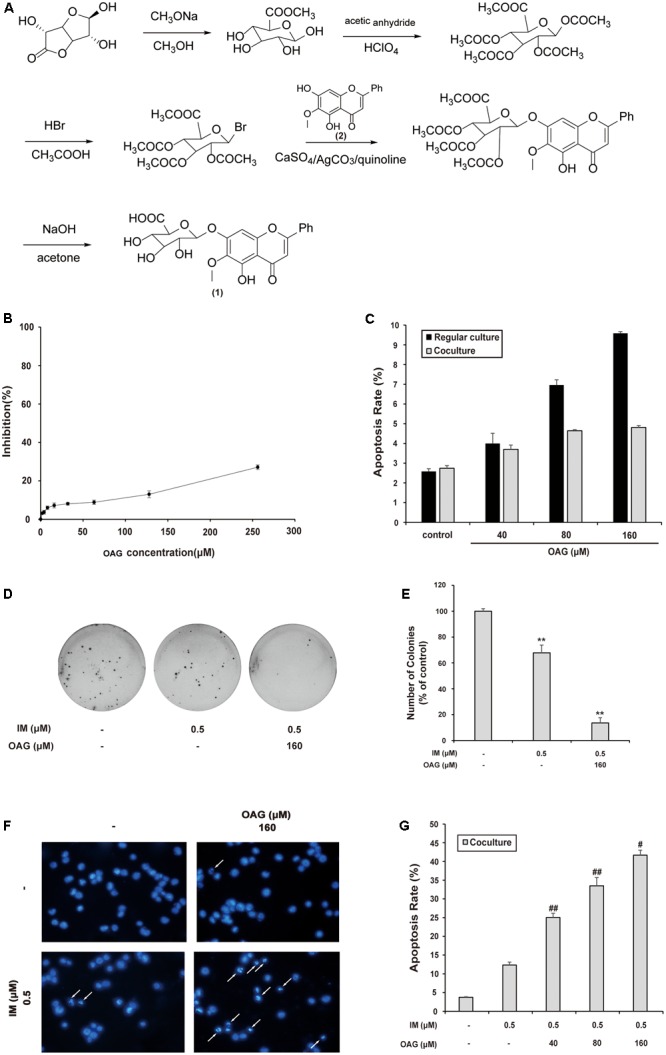
Oroxyloside A (OAG) sensitized CML to IM in co-culture model. **(A)** The synthetic route of OAG. (1) is the molecular structure of OAG (C_22_H_20_O_11_, MW = 460.39). (2) is OA structure (C_16_H_12_O_5_, MW = 284.26). **(B)** K562 cells were treated with various concentration of OAG for 36 h, followed by MTT assay. **(C)** Cells were treated with indicated concentration of OAG for 36 h, and then stained with Annexin V/PI followed by flow cytometry analysis. The apoptotic rates of cells induced by OAG were shown as means ± SD of three independent experiments. **(D–G)** The co-cultured K562 cells were treated as indicated for 36 h, followed by different assay. Representative photos of soft agar colony assay were shown **(D)** and the percentage of colonies was represented as mean values of three experiments ± SD **(E)**. Cell nuclear condensation was pointed out using white arrows in DAPI staining pictures **(F)**. Toxicity of OAG and/or IM was analyzed by Annexin V/PI double-staining assay using flow cytometry. Data are shown as means ± SD for three independent experiments **(G)**. (^∗∗^*p* < 0.01, ^∗^*p* < 0.05 compared with control and ^##^*p* < 0.01, ^##^*p* < 0.05 compared with adjacent group)

Oroxyloside A was synthesized as described in Section “Materials and Methods” part, and the route was shown in **Figure 3A**. We firstly evaluated the toxicity of OAG on K562 cells by MTT assay and Annexin V/PI double-staining assay. As a result, OAG exhibited very low cytotoxic effect on cells (**Figure 3B**), and we choose the concentration of 40, 80, and 160 μM for the following experiments since no severe apoptosis events occurred, especially in co-culture system(**Figure 3C** and Supplementary Figure S4A).

To assess the synergy effect of OAG with IM in co-culture system, we examined the growth and apoptosis of K562 cells treated with IM alone or combined with OAG. As shown in **Figures 3D,E**, the combination of OAG with IM significantly decreased the percentage of colonies to 13.56 ± 4.13%, compared to that of K562 cells treated IM alone (67.80 ± 5.93%) under the condition of co-culturing with HS-5 cells. In addition, OAG synergized with IM to cause K562 cell apoptosis in co-culture model as evaluated by DAPI and Annexin V/PI staining (**Figures 3F,G** and Supplemental Figure S4B). Collectively, the weakly toxic concentrations of OAG could, at least in part, overcome BM microenvironment-induced K562 cell resistance to IM.

### OAG Inhibits Hh Signaling Pathway and Down-regulates BCR-ABL Expression

**FIGURE 4:**
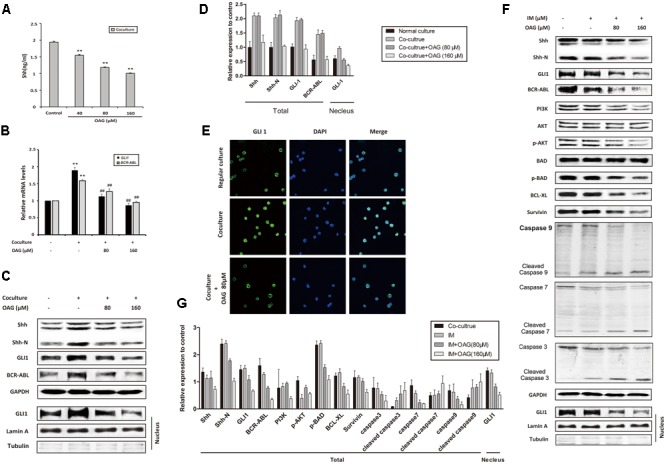
Oroxyloside A inhibited hedgehog pathway and down-regulated BCR-ABL in co-culture system. **(A)** Cells were co-cultured for 24 h and then treated with OAG for another 36 h in a dose-dependent manner. After that, culture supernatants were collected and subjected to ELISA to detect Shh secretion (^∗∗^*p* < 0.01 compared with control). **(B–E)** Regular cultured and co-cultured K562 cells were treated with OAG as indicated. Real-time PCR, western blot and Immunofluorescence were performed for analysis of *GLI1* and *BCR-ABL* mRNA expression **(B)**, Shh, Shh-N, total GLI1, BCR-ABL **(C,D)** and nuclear GLI1 localization **(E)** (^∗∗^*p* < 0.01 compared with regular co-culture and ^##^*p* < 0.01, ^#^*p* < 0.05 compared with control in co-culture). **(F,G)** Co-cultured cells were treated with IM alone or combined with OAG for 36 h as indicated. The whole lysates and nuclear extract were subjected to western blot for evaluating Hh pathway and canonical apoptosis pathway related proteins, as well as nuclear GLI1 expression. GAPDH was used as total protein control, whereas Lamin A and Tubulin was used for evaluating nuclear fractionation efficiency.

As mentioned above, Hh pathway activation and subsequent BCR-ABL up-regulation could be involved in the IM resistance of CML, thus we ask whether OAG could affect Hh signaling in co-culture system. Shh, as a Hh ligand, could activate signaling pathway through paracrine mechanism in CML (Long et al., 2011). **Figure 4A** showed that the amount of Shh was decreased from 1.94 ± 0.02 to 1.00 ± 0.01 ng/ml by OAG in a dose-dependent manner. Besides, both *GLI1* and *BCR-ABL* mRNA expression was promoted under co-culturing condition, whereas OAG treatment suppressed their up-regulation (**Figure 4B**). Consistently, treatment with OAG resulted in protein down-regulation such as Shh, Shh-N, GLI1, and BCR-ABL, as well as GLI1 nuclear export in co-cultured cells (**Figures 4C–E**). Moreover, the addition of OAG combined with IM not only down-regulated the level of BCR-ABL as well as its related proteins, but it also induced cleaved caspases, a well-known events for cell apoptosis, in co-cultured K562 cells (**Figures 4F,G**). These data draw a possible mechanism that OAG could suppress the activation of Hh/BCR-ABL axis, thereby facilitating IM to cause cell death in drug-resistant CML cells.

### OAG Synergizes with IM to Suppress CML Development *In Vivo*

**FIGURE 5:**
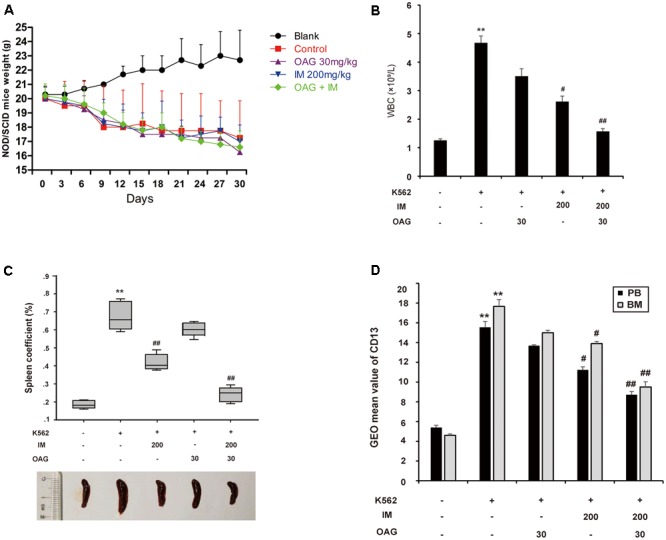
Oroxyloside A potentiated CML repression by IM *in vivo*. **(A)** NOD/SCID mice weight was recorded in all groups every 3 days. **(B–D)** At the end of experiment, all animals were sacrificed and samples were collected to analyze WBC **(B)**, the weight of spleen **(C)** and expression of CD31 in PB and BM **(D)**. The data represent mean values of three experiments ± SD. (^∗∗^*p* < 0.01 compared with blank group and ^##^*p* < 0.01, ^#^*p* < 0.05 compared with control group).

To further support that OAG sensitized CML cells to IM *in vivo*, we inoculated K562 cells into female NOD/SCID mice by intravenous injection as described in Section “Materials and Methods” part. Mice with graft were treated with or without IM (200 mg/kg), together with or without OAG (30 mg/kg). All animals were raised for 4 weeks, and their body weight was monitored every 3 days. As shown in **Figure 5A**, mice transplanted K562 cells showed decreased body weight with days. However, there was no further reduction of body weight in mice treated with drugs compared to those with vehicle treatment, suggesting that drug itself could be safe for chemotherapy. As expected, the amount of white blood cells (WBC) was significantly increased from 1.26 ± 0.06 to 4.68 ± 0.25 × 10^9^/L due to K562 cell transplantation, whereas it was notably decreased to 1.57 ± 0.11 × 10^9^/L by the treatment in combination of IM and OAG (**Figure 5B**). In addition, spleens were weighed to calculate the spleen coefficient. K562 cell graft significantly raised spleen coefficient to 0.68 ± 0.05, which was back to normal range in the presence of IM combined with OAG (**Figure 5C**). Next, the expression of CD13, a surface marker of K562 cells, was analyzed by FACS. As shown in Supplementary Figure S5 and **Figure 5D**, grafted mice treated with OAG and IM together showed significant reduction in CD13 positive cells, suggesting that the combination treatment could eliminate most of K562 cells *in vivo*.

### OAG Inhibits Hh Signaling and BCR-ABL Expression *In Vivo*

**FIGURE 6:**
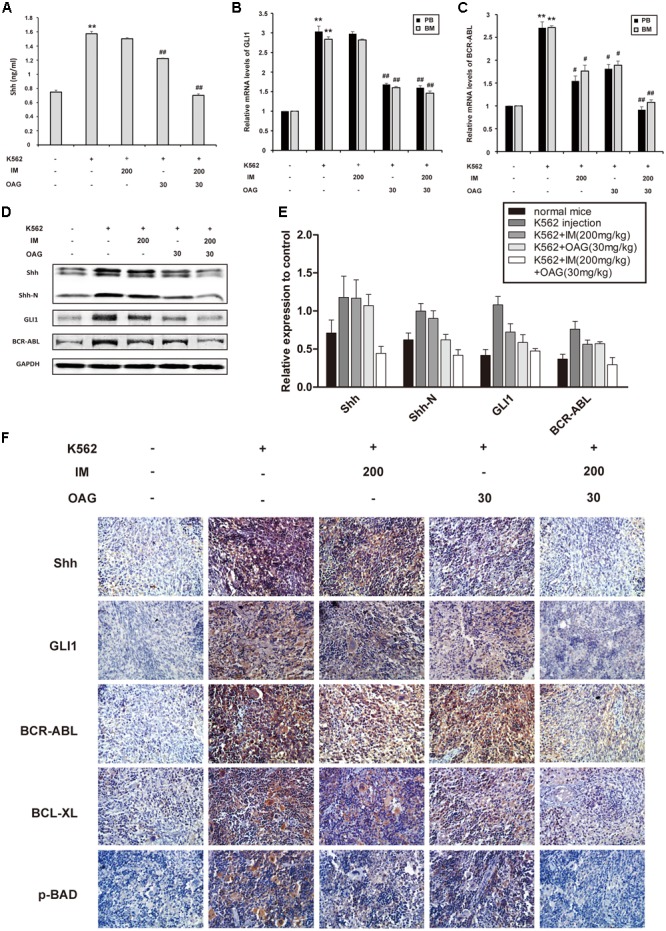
Oroxyloside A suppressed hedgehog signaling and BCR-ABL expression combined with IM treatment *in vivo*. **(A)** The level of Shh cytokine in PB was evaluated by ELISA. **(B)** *GLI1* and **(C)** *BCR-ABL* mRNA levels in PB and BM samples from tree mice of each group were detected by quantitative real-time RT-PCR and fold changes were normalized to GAPDH. The data represented mean values of three experiments ± SD. **(D,E)** Western blot and immunohistochemistry **(F)** was used to examine Shh, Shh-N, GLI1, BCR-ABL and other related proteins in spleen cells. (^∗∗^*p* < 0.01 compared with blank group and ^#^*p* < 0.05, ^##^*p* < 0.01 compared with control group).

Consistent with *in vitro* data, K562 cell inoculation elevated blood Shh for about twofold, whereas OAG alone and its combination with IM could diminish the level of Shh in blood of mice with K562 cell graft (**Figure 6A**). Furthermore, the relative mRNA levels of *GLI1* and *BCR-ABL* in PB and BM were significantly inhibited by combination treatment in inoculated mice (**Figures 6B,C**). In addition, the expression of Shh, Shh-N, GLI1, and BCR-ABL in spleens of control group was up-regulated compared with non-inoculated group, which was significantly suppressed by combination treatment (**Figures 6D–F**). Taken together, the results above suggested that the synergistic effect of IM and OAG against CML cells *in vivo* could be attributed to suppressing Hh pathway and BCR-ABL expression.

## Discussion

Although the development of TKIs played a significant role in Ph(+) leukemia treatment, its resistance has become a major obstacle of success in CML chemotherapy (Volpe et al., 2009). Therefore, we strove to explore a clinically meaningful combination therapy pattern with a new agent to treat CML and prevent relapse. In current study, we showed that the cell co-culture model which mimicked BM microenvironment protected CML cells from IM treatment by up-regulating the Hh pathway and then the BCR-ABL/PI3K/AKT signaling. Furthermore, OAG at low-toxic concentration suppressed Hh pathway, thus sensitizing CML cells to IM *in vitro* and *in vivo*.

As we all know, BM is the source of hematopoietic stem cells (HSC) and LSCs, which generally retain in the quiescent phase and is capable to self-renewal to sustain CML process and induce relapse (Crews and Jamieson, 2013). As previously reported, BM shelter LSCs from drug aggression with complex BM microenvironment containing Cytokines, chemokines, or growth factors secreted by surrounding BMSC (Dazzi et al., 2006). As we found here, co-culture of K562 and HS-5 cells significantly enhanced K562 cell proliferation upon IM treatment. Consistently, the expression of *BCR-ABL* mRNA and levels of BCR-ABL and relative anti-apoptosis and survival proteins was significantly increased in co-cultured cells, which were associated with IM resistance. These results suggested that HS-5 cell-mediated microenvironment rendered K562 cells capability of being non-sensitive to IM treatment.

Previous investigations indicated that the Hh pathway plays an important role in drug resistance of CML (Liao et al., 2012; Zhou et al., 2013). We showed that co-cultured K562 cells harbored elevated Shh, Shh-N, and GLI1 at mRNA and protein level, suggesting that the presence of BMSC could promote the activation of Hh pathway since these proteins played important roles in signal propagation. The activation of Hh pathway was also further proved by the increased nuclear translocation of GLI1, which would initiate the transcription of a large number of target genes. The previous study has demonstrated that BCR-ABL gene could be one of down-stream target of Hh pathway in CML (Liao et al., 2012). As we treated cells with the siRNA specific against GLI1 or GANT-61—a inhibitor of GLI1 nuclear translocation (Merchant et al., 2010), the expression of BCR-ABL correspondingly decreased in co-cultured cells, indicating that Hh pathway activation could up-regulate BCR-ABL in co-cultured K562 cells, which was in agreement with previous report. This interaction of Hh pathway and BCR-ABL signal might explain CML resistance to IM in co-culture system. Undeniably, there might be other pathways involved in BM microenvironment-mediated CML drug resistance, which needs further investigation.

We have previously reported that the oral administration of OA could reverse drug resistance in animal model (Li et al., 2015). As the major metabolite of the bioactive flavonoid OA (Kim et al., 2007; Li et al., 2011; Fong et al., 2014), OAG harbor better water solubility and lower cytotoxicity, and could play multiple functions on cancer cells, which remained unclear. In our studies, we first chose weakly toxic concentrations (40, 80, and 160 μM) of OAG to explore whether it possessed a reverse effect on drug resistance of CML. Subsequently, we proved that OAG could significantly overcome IM resistance of K562 cells via inhibiting proliferation and promoting apoptosis. In addition, the alternation of several leukemia indexes such as WBC proportion in PB, CD13 in PB and BM, and the spleen weights of NOD/SCID mice bearing K562 cells further supported the synergistic effect of IM and OAG against leukemic cells. As expected, OAG dramatically inhibited cytoplasmic Shh and its secretion by K562 cells, suggesting that OAG could block autocrine Hh pathway which has been implicated in CML (Long et al., 2011). Although the expression of canonical apoptosis pathway mediators-caspases was marginally altered (data not shown), the vital mediators of Hh pathway and BCR-ABL, as well as the nuclear translocation of Gli1 in the co-culture model were all suppressed by the addition of OAG (**Figures 4C,F**). Moreover, OAG together with IM treatment attenuated Hh and BCR-ABL down-stream signal, such as PI3K/AKT pathway, and p-BAD, BCL-XL, and Survivin protein expression, eventually triggering cell apoptosis pathway compared with IM treatment alone. These data suggest OAG itself exhibited low cytotoxicity, however, could enhance the sensitivity of CML cells to IM therapy by suppressing Hh pathway and then BCR-ABL expression.

Collectively, co-culturing with HS-5 cells resulted in the aberrant activation of Hh pathway and subsequent BCR-ABL overexpression in K562 cells which could contribute to IM resistance of CML, whereas OAG sensitized K562 cells to IM treatment through inhibiting Hh pathway and then BCR-ABL expression *in vitro* and *in vivo*. Suppression of Hh signaling and subsequent down-regulation of BCR-ABL would be apparently beneficial for apoptosis caused by IM, eventually facilitating CML treatment. Through mimicking BM microenvironment, our study provided direct evidence that OAG could overcome IM resistance of K562 cells.

## Author Contributions

Conceived and designed the experiments: QG, LZ, ZL, XZ, and YL. Performed the experiments: XZ, YL, LL, SH, ZL and YZ. Analyzed the data: XZ, YL, YZ, ZL, and LZ. Contributed reagents/materials/analysis tools: ZL, LZ, XZ, and YL. Wrote the manuscript: XZ and YL. All authors reviewed the manuscript.

## Conflict of Interest Statement

The authors declare that the research was conducted in the absence of any commercial or financial relationships that could be construed as a potential conflict of interest.
