# Supplementary material for: Oroxyloside A Overcomes Bone Marrow Microenvironment-Mediated Chronic Myelogenous Leukemia Resistance to Imatinib via Suppressing Hedgehog Pathway
Source: Front Pharmacol. 2017 Aug 11;8:526. doi: 10.3389/fphar.2017.00526 (PMC5554535; doi:10.3389/fphar.2017.00526)
Supplement: Supplementary file 1 [file Data_Sheet_1.pdf]

**Sample Name**		**Position**	P1-D7	**Instrument Name**	Instrument 1	**User Name**	
**Inj Vol**	2	**InjPosition**		**SampleType**	Sample	**IRM Calibration Status**	Success
**Data Filename**	XZL-QCZSG-p.d	**ACQ Method**		**Comment**		**Acquired Time**	9/15/2014 8:16:45 PM

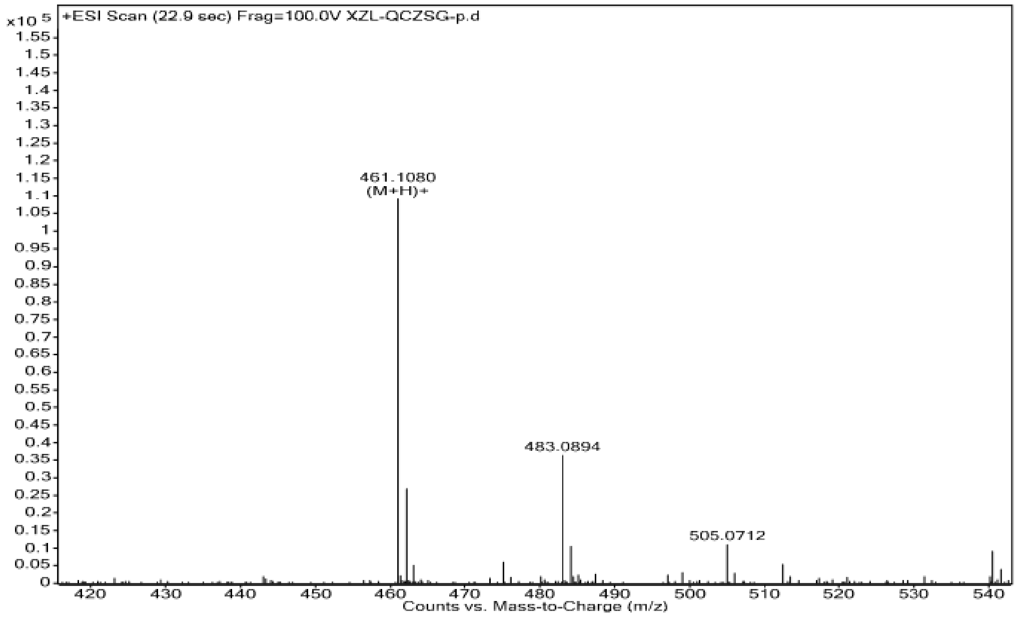

MS Formula Results: + Scan (22.9 sec) (XZL-QCZSG-p.d)

m/z	Ion	Formula	Abundance
461.108	(M+H)+	C22 H21 O11	109079.3

Best	Formula (M)	Ion Formula	Calc m/z	Score	Cross Score	Mass	Calc Mass	Diff (ppm)	Abs Diff (ppm)	Abund Match	Spacing Match	Mass Match	m/z	DBE
☑	C22 H20 O11	C22 H21 O11	461.1078	99.35		460.1007	460.1006	-0.3	0.3	99.58	97.97	99.91	461.108	13
☐	C23 H16 N4 O7	C23 H17 N4 O7	461.1092	94.98		460.1007	460.1019	2.6	2.6	97.03	95.65	93.41	461.108	18
☐	C19 H12 N10 O5	C19 H13 N10 O5	461.1065	92.55		460.1007	460.0992	-3.26	3.26	99.5	89.59	89.86	461.108	19

Supplementary Fig S1. HRMS Analysis

HRMS (ESI): m/z, calculated for $C_{22}H_{20}O_{11}$, 461.1078 $(M + H)^+$, found 461.1080.

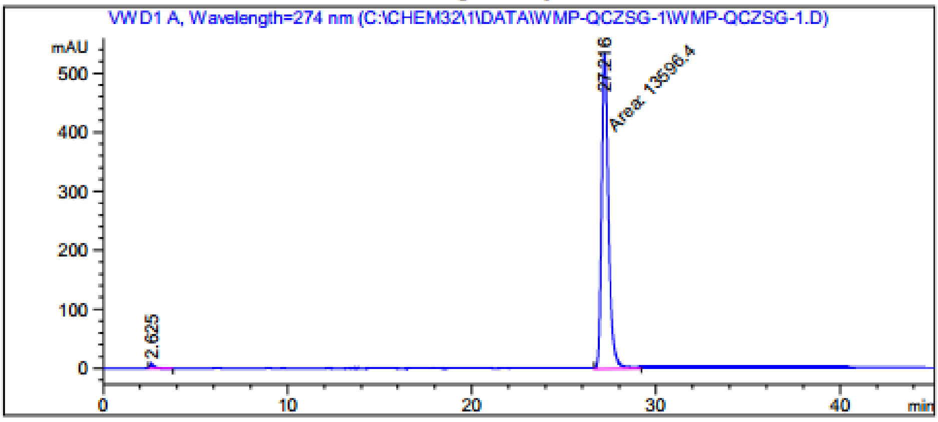


```
========================================================================
                         Area Percent Report
========================================================================

Sorted By             :      Retention Time
Multiplier:                  :       1.0000
Dilution:                    :       1.0000
Sample Amount:               :      1.00000   [Mg/ML]    (not used in calc.)
Use Multiplier & Dilution Factor with ISTDs

Signal 1: VWD1 A, Wavelength=274 nm

Peak RetTime Sig Type    Area       Height      Area
  #   [min]              [mAU*s]     [mAU]        %
----|-------|---|----|----------|----------|--------|
   1   2.625   1  VB     99.85452    7.95564   0.7291
   2  27.216   1  MM   1.35964e4   533.74548  99.2709
```

Supplemental Fig. S2 HPLC Analysis

HPCL Purity>99.0 %

The purity (> 99.0%) of final compounds is verified by the HPLC study performed on Agilent C18 (4.6 mm×150 mm, 3.5 μm) column using a mixture of solvent methanol/water at the flow rate of 1.0 mL/min and peak detection at 274 nm under UV.

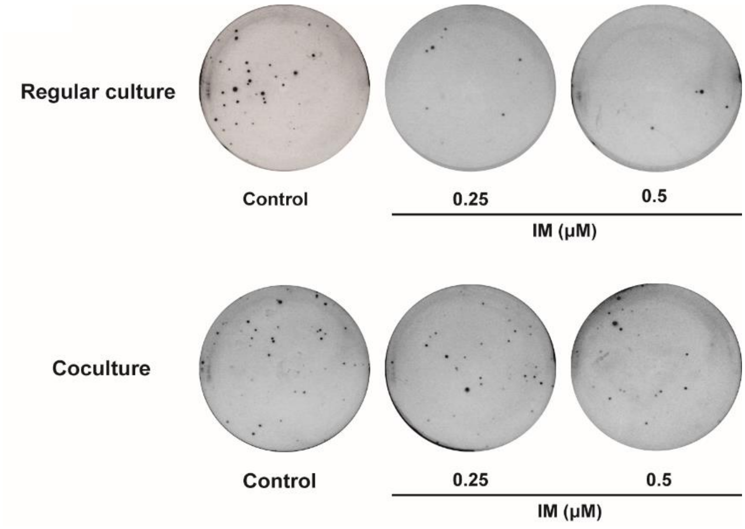

Supplemental Fig. S3A Soft agar colony formation experiment was performed to ascertain the IM inhibition effect.

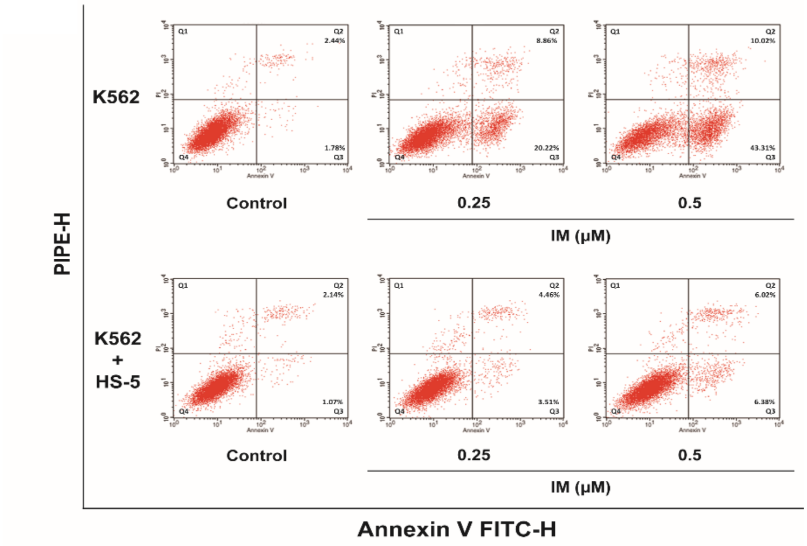

Supplemental Fig. S3B Annexin V/PI double-staining assay of the toxicity of IM in coculture or not analyzed by flow cytometry.

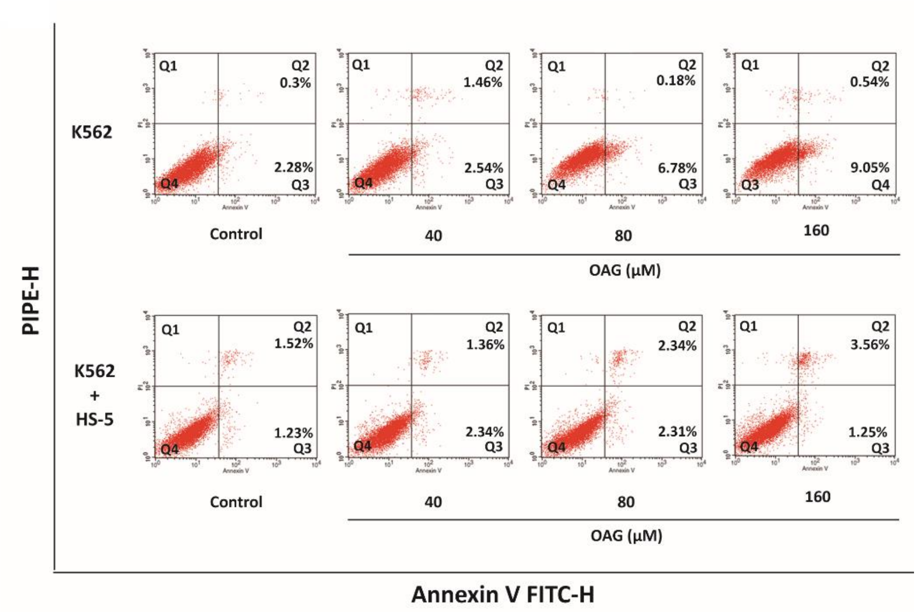

Supplemental Fig. S4A AnnexinV/PI double-staining assay of the intrinsic toxicity of OAG (40, 80, and 160μM) was analyzed by flow cytometry.

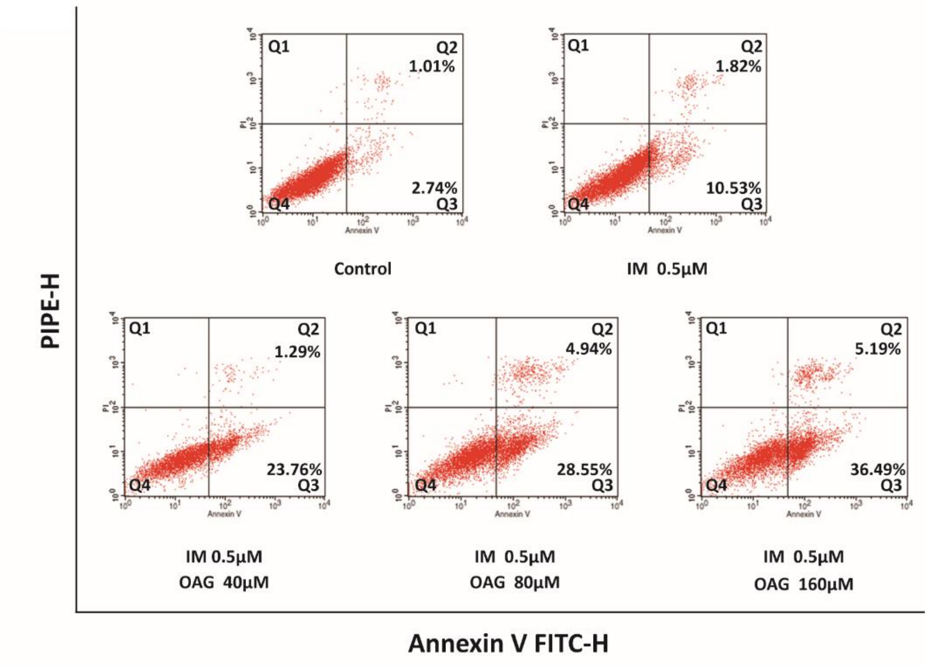

Supplemental Fig. S4B AnnexinV/PI double-staining assay of the toxicity of OAG (40, 80, and 160μM) and/or IM (0.5μM) was analyzed by flow cytometry.

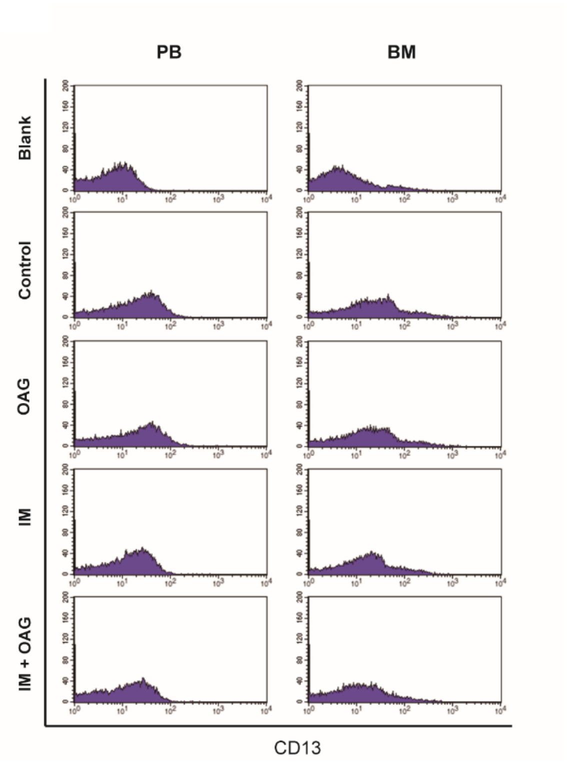

Supplemental Fig. S5 The expression of CD13 in PB and BM samples from tree mice of each group.
